# Stable Isotope Analysis Can Potentially Identify Completely-Digested Bloodmeals in Mosquitoes

**DOI:** 10.1371/journal.pone.0002198

**Published:** 2008-05-21

**Authors:** Jason L. Rasgon

**Affiliations:** The W. Harry Feinstone Department of Molecular Microbiology and Immunology, and the Johns Hopkins Malaria Research Institute, Bloomberg School of Public Health, Johns Hopkins University, Baltimore, Maryland, United States of America; University of Oxford, United Kingdom

## Abstract

**Background:**

Vertebrate bloodfeeding is a critical component of a mosquito's ability to transmit pathogens that cause diseases such as malaria, dengue fever and viral encephalitis. Due to degradation by the digestive process, current methods to identify mosquito bloodmeal sources are only useful for approximately 36 hours post-feeding. A critical need exists for technologies to extend this window and gain a more complete picture of mosquito feeding behavior for epidemiological studies. Stable isotopes are useful for investigating organism feeding behavior because the isotopic ratio of an organism's tissues reflects that of the material it ingests.

**Methodology/Principal Findings:**

Proof-of-principle data indicates that after bloodfeeding, *Aedes albopictus* mosquitoes acquire diagnostic Carbon and Nitrogen stable isotope profiles from their vertebrate hosts that can be accurately identified one week post-feeding, approximately 4 days after the entire bloodmeal has been digested. Total C/N ratio served as a biomarker marker for bloodfeeding (*P*<0.02), while δN was the most informative variable which could distinguish between unfed, chicken-fed and human-fed mosquitoes (*P*<0.01). By plotting C/N vs. δN, all feeding treatments could be identified in a double-blind analysis.

**Conclusions/Significance:**

These proof-of-principle experiments indicate that analysis of stable isotopes can be used to distinguish bloodfed from unfed mosquitoes, and also distinguish between different vertebrate bloodmeal sources even after all blood has been digested. The development of stable isotope-based assays for mosquito bloodmeal identification may be a powerful tool to investigate mosquito feeding ecology and the dynamics of vector-borne pathogens.

## Introduction

Re-emerging vector-borne diseases, such as malaria, dengue and West Nile viral encephalopathy are a significant public health threat. For example, human malaria, caused by 4 protozoan parasites in the genus *Plasmodium*, infects up to 500 million people per year and is responsible for almost 3 million deaths annually [1]. Dengue viral infections cause more human morbidity and mortality than any other arthropod-borne virus disease [2], [3]. Since its introduction in 1999, West Nile Virus (WNV) has spread completely across the contiguous United States and has been responsible for over 20,000 confirmed human cases with over 1,000 deaths [4].

Vector-borne pathogens are dependent on vector insects for propagation from one vertebrate host to the next. Vector insects usually acquire these pathogens from feeding on an infected host, and transmit the pathogen to a naive host during subsequent feeding events. Mosquito bloodfeeding behavior is a very significant component of pathogen transmission and determinant of disease epidemiology. The tendency for certain mosquito vectors to feed primarily on humans is also a major factor driving the transmission of dengue and malaria [5]. It is important to accurately identify the sources of mosquito bloodmeals to determine if mosquitoes are feeding on epidemiologically relevant hosts. This information can be used to selectively target control efforts to epidemiologically relevant vector species and to conduct proactive risk assessment on the vector potential of mosquito populations prior to pathogen introduction. In addition, one would like to identify mosquitoes that feed on multiple host types. Mosquitoes that tend to feed on multiple host species have the potential to act as bridge vectors, occasionally transferring pathogens from the reservoir host/maintenance cycle to humans or domestic animals. For example, West Nile Virus (WNV) is primarily an infection of birds, but can be transferred to humans or horses by mosquitoes that feed on both birds and mammals [6]–[10].

Historically, immunological techniques such as hemagglutination or ELISA have been used to identify the vertebrate species that the mosquitoes fed upon [11]–[14]. Recently, the more sensitive polymerase chain reaction (PCR) has been used for identifying the species and/or individual upon whom the mosquito fed [15]–[19]. While these methods are useful for studying mosquito bloodfeeding behavior, they all depend on the presence of undigested blood in the mosquito midgut. As such, they have a very narrow window in which they can be utilized. After approximately 36 hours, the bloodmeal is sufficiently digested to make identification by immunological or PCR-based techniques highly problematic [16]. To accurately study mosquito bloodfeeding behavior, one would like a method to identify vertebrate bloodmeal sources even when no detectable blood remains in the mosquito.

Different isotopes of an element have different numbers of neutrons and hence, a different atomic mass. For example, the most abundant carbon isotopes are carbon-12 (^12^C) containing 6 protons, 6 electrons and 6 neutrons; carbon-13 (^13^C), containing 6 protons, 6 electrons and 7 neutrons; and carbon-14 (^14^C), containing 6 protons, 6 electrons and 8 neutrons. Too many or too few neutrons cause some isotopes to be unstable (^14^C for example), and these ultimately form stable products by radiodecay. Other isotopes which have stable combinations of neutrons and protons (e.g. ^12^C and ^13^C) do not decay, and are referred to as stable isotopes. Stable isotopes in biological samples are can be analyzed by gas isotope-ratio mass spectroscopy, in which samples are burned, converted into a gas, ionized, and separated in a magnetic field according to their mass. The intensity of each mass-sorted ion beam is then measured, allowing for quantification of isotope values [20]–[21].

Stable isotopes of carbon, nitrogen, sulfur, oxygen, and hydrogen are most commonly analyzed for ecological research. Studies have shown that the stable isotopic ratio of an organism's tissues reflects the isotopic ratio of the material it ingests (by eating, drinking or breathing). One can thus compare the isotopic ratio of an organism and make inferences about what it has ingested [20], [22]–[25]. If stable isotope analysis protocols were developed to address questions directly related to mosquito feeding behavior, it might be possible to qualitatively and quantitatively reconstruct the lifetime feeding history of individual wild-caught mosquitoes. In this paper, we demonstrate in proof-of-principle experiments that stable isotope analysis can be used to identify the vertebrate source of mosquito bloodmeals after complete digestion.

## Results

**Figure 1:**
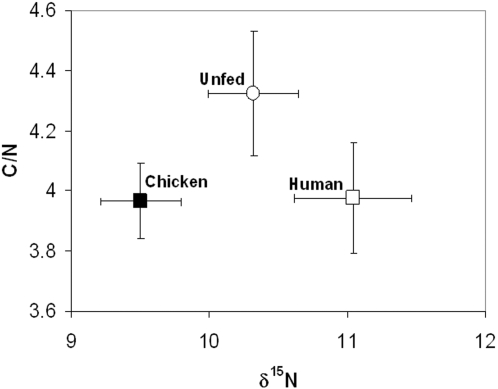
Plot of C/N ratio vs. δN for feeding treatments. *N* = 10 mosquitoes/treatment. Plots are sample means±95% confidence intervals.

Analysis of stable isotopes was able to distinguish between feeding treatments (Figure 1). Five-day-old mated adult female *Aedes albopictus* (Houston strain) mosquitoes were either held without being bloodfed (Un; unfed), or allowed to feed for 15 minutes on a lightly-restrained chick (Ch; chicken) or on the arm of the author (Hu; human). Mosquitoes were held for one week to allow for complete digestion of the bloodmeal, after which they were placed in ethanol, coded and analyzed for 5 variables: (δN, δC, %N, %C and C/N) in a double-blind manner (see Materials and Methods for details). Wilks'-Lambda multivariate analysis of variance (MANOVA) indicated that stable isotope profiles of at least one feeding treatment differed significantly (*P*<0.0001). Fishers PLSD pairwise comparisons indicated that there were significant differences among feeding treatments for δN (Ch vs. Hu, *P*<0.0001; Ch vs. Un, *P* = 0.0035; Hu vs. Un, *P* = 0.0082), %N (Ch vs. Un, *P* = 0.0058), and C/N (Ch vs. Un, *P* = 0.0088; Hu vs. Un, *P* = 0.0106). C/N ratio can distinguish between bloodfed and unfed mosquitoes but not between host types and is thus potentially useful as a biomarker for bloodfeeding. δN was the most informative variable and could distinguish unfed, chicken-fed and human-fed mosquitoes. By plotting C/N vs. δN, good discrimination between all three treatments was observed (Fig. 1).

## Discussion

These proof-of-principle experiments indicate that analysis of stable isotopes can be used to distinguish bloodfed from unfed mosquitoes, and also distinguish between different vertebrate bloodmeal sources even after all blood has been digested. In this preliminary study, 2 different host types were examined at a single time-point for 2 isotopes. Field situations will likely be more complicated, with multiple potential hosts present in the environment. Analysis of other isotopes (such as Hydrogen, Oxygen or Sulfur) may improve the resolution of bloodmeal identification, especially in situations of many potential host species. Initial stable isotope profiling will have to be performed against all potential hosts, and temporal-spatial variation in stable isotope profiles within populations and within individual mosquitoes taken into account. Multiple or sequential bloodfeeding events may also complicate profiles, and should be investigated. It should be noted that these limitations are not unique to stable isotope-based profiling techniques, but are important for other bloodmeal identification assays such as PCR.

For complex feeding systems, it may be beyond the resolution of stable isotope profiling to unambiguously resolve all hosts. While stable isotope profiling is more sensitive than PCR in identifying digested bloodmeals, it is likely less specific. To gain the most complete picture of mosquito feeding habits, stable isotope profiling would be best used in concert with PCR, with PCR utilized on fresh bloodmeals to gain a snapshot picture of present feeding habits, and stable isotopes utilized on specimens unsuitable for PCR (such as gravid individuals or mosquitoes with digested or no observable bloodmeal) to reconstruct the history of previous feeding events.

Because collected mosquitoes can be simply placed into ethanol and stored until processed, stable isotope analysis may be useful for those working under field conditions in developing countries. If outsourcing analysis to a commercial laboratory, ethanol-preserved samples can be directly shipped to the laboratory to be processed onsite. Samples can also be processed by the investigators if a good microbalance is available to accurately weigh specimens–samples are simply removed from the ethanol, dried, weighed, ground and placed into tin capsules. Costs can range from $4–$30 per sample, depending on whether the investigators analyze the samples themselves or outsource analysis, the amount of sample processing performed by the investigator in the case of outsourcing (drying, weighing, grinding, and/or encapsulation), the specific isotopes examined and the number of samples analyzed.

The successful development of stable isotope-based assays for identification of mosquito bloodmeals has the potential to be a powerful new tool to study vector bloodfeeding ecology. One validated under field conditions, this technique will stimulate novel efforts to address critical questions related to mosquito ecology, feeding behavior and the dynamics of vector-borne pathogens.

## Materials and Methods

### Mosquitoes

The *Aedes albopictus* Houston strain was used for experiments. Mosquito colonies were reared in 30 cm cubic screen cages in a walk-in insectary at 27°C, 90% RH on a 16∶8 hr light∶dark cycle. Larvae were fed a 1∶2∶2 mix of ground fish food (Tetramix), rabbit pellets and bovine liver powder. Adult mosquitoes were allowed access to a cotton pad soaked in 10% sucrose solution as a carbohydrate source.

### Feeding

After emergence, adult mosquitoes were allowed to mate for 5 days, after which they were either held without being bloodfed (Un; unfed), or allowed to feed for 15 minutes on a lightly-restrained chick (Ch; chicken) (protocol AV04H369) or on the arm of the author (Hu human) according to established informed consent procedures. After bloodfeeding, engorged mosquitoes were separated and held for an additional 7 days to allow for complete digestion of the bloodmeal. Unfed mosquitoes were held for a similar amount of time. Fed mosquitoes were analyzed as gravid females to maximize the probability of detecting deposited isotopes in tissues. After one week, mosquitoes were placed into 100% ethanol for 2 weeks.

### Sample analysis

All samples were coded by a third party and analyzed in a double-blind manner. Samples were removed from ethanol, dried, ground, weighed and placed into tin analysis capsules (Costech Analytical Inc.). Samples were analyzed using a Thermo Finnigan DELTA^plus^ Advantage gas isotope-ratio mass spectrometer configured with a CONFLO III for automated continuous-flow analysis using a Carlo Erba NC2100 Elemental Analyzer (Thermo Electron Corporation). For each sample, data were collected for 5 variables: δN, δC, %N, %C and C/N. Data were normalized using internationally-accepted isotope standards: Air for Nitrogen and Vienna Peedee Belemnite for Carbon. Stable isotope ratios were expressed in δ notation as parts per thousand deviations according to the equation
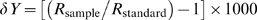
(1)Where *Y* is the element of interest (N or C) and *R* is the corresponding ratio of ^15^N∶^14^N or ^13^C∶^12^C.

### Statistical analysis

Because the dependent variables (δN, δC, %N, %C and C/N) may be correlated, significant differences between feeding treatments were assessed by multivariate analysis of variance (MANOVA). Significance of individual factors was assessed by individual analysis of variance (ANOVA) tests. For significantly-different factors, pair-wise differences between treatments were determined by Fisher's Protected Least Significant Difference (PLSD).

## References

[1] Snow RW, Guerra CA, Noor AM, Myint HY, Hay SI (2005). The global distribution of clinical episodes of *Plasmodium falciparum* malaria.. Nature.

[2] Monath TP (1994). Dengue: the risk to developed and developing countries.. Proc Natl Acad Sci USA.

[3] Kuno G (1995). Review of the factors modulating dengue transmission.. Epidemiol Rev.

[4] CDC (2007). http://www.cdc.gov/ncidod/dvbid/westnile.

[5] Dye C (1992). The analysis of parasite transmission by blood-sucking insects.. Annu Rev Entomol.

[6] Andreadis TG, Anderson JF, Vossbrinck CR, Main AJ (2004). Epidemiology of West Nile virus in Connecticut: a five-year analysis of mosquito data 1999–2003.. Vector Borne Zoonotic Dis.

[7] Gratz NG (2004). Critical review of the vector status of *Aedes albopictus*.. Med Vet Entomol.

[8] Kilpatrick AM, Kramer LD, Campbell SR, Alleyne EO, Dobson AP (2005). West Nile virus risk assessment and the bridge vector paradigm.. Emerg Infect Dis.

[9] Kutz FW, Wade TG, Pagac BB (2003). A geospatial study of the potential of two exotic species of mosquitoes to impact the epidemiology of West Nile virus in Maryland.. J Am Mosq Control Assoc.

[10] Sardelis MR, Turell MJ, O'Guinn ML, Andre RG, Roberts DR (2002). Vector competence of three North American strains of *Aedes albopictus* for West Nile virus.. J Am Mosq Control Assoc.

[11] Beier JC, Perkins PV, Wirtz RA, Koros J, Diggs D (1988). Bloodmeal identification by direct enzyme-linked immunosorbent assay (ELISA), tested on *Anopheles* (Diptera: Culicidae) in Kenya.. J Med Entomol.

[12] Chow E, Wirtz RA, Scott TW (1993). Identification of blood meals in *Aedes aegypti* by antibody sandwich enzyme-linked immunosorbent assay.. J Am Mosq Control Assoc.

[13] Gomes LA, Duarte R, Lima DC, Diniz BS, Serrao ML (2001). Comparison between precipitin and ELISA tests in the bloodmeal detection of *Aedes aegypti* (Linnaeus) and *Aedes fluviatilis* (Lutz) mosquitoes experimentally fed on feline, canine and human hosts.. Mem Inst Oswaldo Cruz.

[14] Tempelis CH, Lofy MF (1963). A Modified precipitin method for identification of mosquito bloodmeals.. Am J Trop Med Hyg.

[15] Boakye DA, Tang J, Truc P, Merriweather A, Unnasch TR (1999). Identification of bloodmeals in haematophagous Diptera by cytochrome B heteroduplex analysis.. Med Vet Entomol.

[16] Chow-Shaffer E, Sina B, Hawley WA, De Benedictis J, Scott TW (2000). Laboratory and field evaluation of polymerase chain reaction-based forensic DNA profiling for use in identification of human blood meal sources of *Aedes aegypti* (Diptera: Culicidae).. J Med Entomol.

[17] Lee JH, Hassan H, Hill G, Cupp EW, Higazi TB (2002). Identification of mosquito avian-derived blood meals by polymerase chain reaction-heteroduplex analysis.. Am J Trop Med Hyg.

[18] Kent RJ, Norris DE (2006). Identification of mammalian blood meals in mosquitoes by a multiplexed polymerase chain reaction targeting cytochrome B.. Am J Trop Med Hyg.

[19] Mukabana WR, Takken W, Knols BG (2002). Analysis of arthropod bloodmeals using molecular genetic markers.. Trends Parasitol.

[20] Wada E, Mizutani H, Minagawa M (1991). The use of stable isotopes for food web analysis.. Crit Rev Food Sci Nutr.

[21] Werner RA, Brand WA (2001). Referencing strategies and techniques in stable isotope ratio analysis.. Rapid Comm Mass Spectro.

[22] Gaye-Siessegger J, Focken U, Muetzel S, Abel H, Becker K (2004). Feeding level and individual metabolic rate affect delta 13C and delta 15N values in carp: implications for food web studies.. Oecologia.

[23] Grupe G, Kruger HH (1990). Feeding ecology of the stone and pine marten revealed by element analysis of their skeletons.. Sci Total Environ.

[24] Hobson KA, Bowen GJ, Wassenaar LI, Ferrand Y, Lormee H (2004). Using stable hydrogen and oxygen isotope measurements of feathers to infer geographical origins of migrating European birds.. Oecologia.

[25] Iko WM, Kester CL, Bern CR, Stendell RC, Rye RO (2003). Isotope variations in white-tailed kites from various habitats in California: possible limitations in assessing prey utilization and population dynamics.. Isotopes Environ Health Stud.

